# Terahertz Spectroscopy for Non-Destructive Solid-State Investigation of Norfloxacin in Paper Tablets after Wet Granulation

**DOI:** 10.3390/pharmaceutics15071786

**Published:** 2023-06-21

**Authors:** Lara Heidrich, Ayat Abdelkader, Jan Ornik, Enrique Castro-Camus, Cornelia M. Keck, Martin Koch

**Affiliations:** 1 Department of Physics and Material Sciences Center, Philipps-Universität Marburg, Renthof 5, 35032 Marburg, Germany; 2 Department of Pharmaceutics and Biopharmaceutics, Philipps-Universität Marburg, Robert-Koch-Str. 4, 35037 Marburg, Germany; 3 Assiut International Center of Nanomedicine, Al-Rajhi Liver Hospital, Assiut University, Assiut 71515, Egypt

**Keywords:** oral drug delivery, granulation, solid-state characterization, X-ray powder diffraction, terahertz time-domain spectroscopy

## Abstract

(1) Background: Amorphous drug systems are an intensively studied approach to overcome the insufficient bioavailability of poorly soluble drugs. Here, paper tablets were studied, which were made from cellulose-based paper matrices loaded with norfloxacin. Moreover, wet granulation was introduced as an additional processing step for improving the flowability of the solids, which is necessary when considering production on an industrial scale. (2) Methods: The possible impact of the wet granulation on the crystallinity of norfloxacin was studied by examining granulated and non-granulated samples. Crystallinity investigations were performed using X-ray powder diffraction (XRD) and terahertz time-domain spectroscopy (THz TDS). (3) Results: THz TDS allowed for a more straightforward crystallinity assessment than XRD. Moreover, using THz TDS, it was possible to detect minor changes in the crystallinity of the API after the granulation, whereas this was not possible with the XRD analysis. (4) Conclusions: THz TDS results indicate a partial crystallization of norfloxacin due to the wet granulation. Depending on the formulation, THz TDS can serve as a beneficial and advantageous tool to determine the crystallinity of an API.

## 1. Introduction

Oral ingestion is the most preferred route of drug delivery because of various benefits, such as ease of administration, cost effectiveness, and high patient compliance [1]. However, one major challenge in the design of oral dosage forms for many new chemical entities is to overcome their poor aqueous solubility to improve their bioavailability [2]. To enhance the poor aqueous solubility of an active pharmaceutical ingredient (API), various approaches have been utilized, including nanoemulsions, micelles formation, lipid-based formulations, solid dispersions, self-emulsifying drug delivery systems, nanocrystals and inorganic nanocarriers [3,4,5]. As a novel drug delivery system, the smartFilms technique has been demonstrated recently [6]. In this approach, cellulose-based paper matrices are used, in which the API is loaded in an amorphous state. Thus, the solubility and dissolution rate can be enhanced as an amorphous drug exhibits improved solubility in comparison to its crystalline counterpart [7].

The production of smartFilms includes three main steps: first, the dissolution of the API in an appropriate (organic) solvent; second, the application of the solution on a cellulose-based paper matrix; and, finally, the drying of the obtained smartFilms. The resulting smartFilms can be easily transferred into convenient oral dosage forms, such as capsules or tablets, fulfilling the requirements of the European Pharmacopoeia [8,9,10]. The key problem of amorphous formulations is the physical instability of the amorphous state which can lead to recrystallization and consequently unpredictable and decreased solubility and, thus, insufficient bioavailability [2]. The stabilization mechanism in smartFilms is not fully elucidated. It is suggested that the pores and fibers of the cellulose-based smartFilm matrix prevent the recrystallization of the API due to their three-dimensional structure and/or intermolecular interactions [6,11].

So far, the preparation of smartFilms and tablets made from the paper has been mainly investigated on a laboratory scale and industrial large-scale production of paper tablets was not possible due to the poor flowability of the paper cutouts [12]. To overcome this, i.e., to transform smartFilms into solids with appropriate flowability for high-speed tablet manufacturing, wet granulation has been recently used to produce granules from the paper pieces [12,13,14]. Moreover, the processing of granules produced by wet granulation was investigated in several studies, showing further advantages such as improved compressibility and uniform drug distribution [15,16,17]. The manufacture of granules involves, first, the addition of a granulation liquid (i.e., water) to a blend of paper (i.e., smartFilms) and sucrose powder to promote agglomeration into granules. Afterwards, the formed granules are dried and processed further [12]. Indeed, within these steps, the API is exposed to water and, if necessary, high temperatures for drying purposes. Both factors can affect the stability of the amorphous API and might result in partial drug dissolution, phase transformation, or recrystallization [18,19]. Since the crystalline state of an API can affect its solubility and consequently its bioavailability, it is mandatory to control the occurrence of a phase transformation of the API within the formulation [7,20].

Traditionally, X-ray powder diffraction (XRD) is the gold standard in solid-state characterization. It has been established for decades and, together with differential scanning calorimetry (DSC), is recommended for solid-state analysis [20]. In addition, other spectroscopic techniques have been utilized to study the crystallinity of pharmaceutical ingredients, such as mid- and near-infrared spectroscopy, Raman spectroscopy, solid-state nuclear magnetic resonance, and terahertz (THz) time-domain spectroscopy (TDS) [21]. THz TDS uses electromagnetic waves in a frequency region that is located between infrared and microwaves. Typical THz TDS instruments enable the investigation in the frequency range from 0.1 to 4 THz, approximately [22]. It is a fast and non-destructive method that can be used in several research areas, such as medicine, engineering, material sciences and chemistry [22,23,24,25]. In reference [26] a comprehensive overview of THz science and technology as well as numerous fields of applications is given. In comparison to X-rays, THz radiation is non-ionizing because of the low photon energy (meV range). Phonon lattice modes can be probed directly, which makes THz TDS a valuable tool for solid-state analysis [27]. Since THz radiation excites intermolecular rather than intramolecular motions, THz TDS can provide information on the collective behavior of the molecules, i.e., in organic crystals [28]. Thus, the distinction between different polymorphs and crystalline states of a substance, including many APIs, is possible. The crystalline form of an API can lead to distinct absorption peaks in the corresponding THz spectra, whereas only a broad, featureless absorption can be found for its amorphous form [29,30]. In the last years, various applications of THz TDS in the fields of chemistry and pharmaceutics have been established, e.g., monitoring the crystallization process from a solution, multicomponent chemical imaging of tablets, or measuring the molecular mobility in amorphous pharmaceuticals [31,32,33]. Moreover, it is possible to quantify the degree of crystallinity of an API in pharmaceutical formulations [8,34]. Also, THz TDS has already been used to study smartFilms and paper tablets along with XRD and DSC. While DSC failed to determine the crystallinity of tartaric acid in the formulations, XRD and THz TDS led to similar results [8]. However, recent studies showed that THz TDS can provide deeper insights into the solid-state properties of pharmaceutical formulations and can lead to a detailed picture of correct crystalline structures over the gold standard, XRD [35,36].

This work focuses on studying tablets made from norfloxacin-loaded paper granules. Norfloxacin is a synthetic antibacterial fluoroquinolone and is classified as BCS class IV because of its low solubility in aqueous media and low permeability [37,38]. The pharmaceutical properties (i.e., the tablet thickness, mass uniformity, friability, resistance to crushing, disintegration, and content uniformity) as well as the dissolution profile and bioactivities of paper tablets loaded with norfloxacin have been investigated previously [13]. smartFilm tablets showed an enhanced dissolution rate and improved antibacterial activity in vitro and in an ex vivo infected model in comparison to their physical mixture tablets. Here, we utilize XRD and THz TDS to investigate the solid-state of norfloxacin in tablets made from smartFilms. We compare the suitability of both methods to study the crystalline state of norfloxacin in the formulations as well as possible changes in the crystallinity due to the granulation of smartFilms. Additionally, the crystallinity of norfloxacin in the samples is assessed quantitatively based on the data acquired by THz TDS.

## 2. Materials and Methods

### 2.1. Materials

Norfloxacin (95%, CAS number: 70458-96-7) was acquired from abcr GmbH (Karlsruhe, Germany) and used as received. As a paper matrix, commercially available cellulose-based paper (Soft & Sicher, dm-drogerie markt GmbH + Co. KG, Karlsruhe, Germany) was utilized. Sucrose (99.5%) was purchased from Carl Roth GmbH + Co. KG (Karlsruhe, Germany). Purified water was obtained from a PURELAB Flex 2 (ELGA LabWater, Veolia Water Technologies GmbH, Celle, Germany).

### 2.2. Sample Preparation

#### 2.2.1. Pure Substances

For XRD analysis, norfloxacin and sucrose were studied as raw bulk materials. For THz TDS all samples were investigated as tablets. Therefore, ca. 120–200 mg of pure norfloxacin or sucrose were pressed into tablets with a hydraulic press (Model No. GF-10B Cl. 1.0, Enerpac, Menomonee Falls, WI, USA) by applying a compression force of 30 kN for 90 s. The resulting tablets had a thickness of 0.8–1.5 mm and a diameter of approximately 13 mm.

#### 2.2.2. Physical Mixtures

To verify if the devices used were adequately sensitive to detect the crystalline ingredients, physical mixtures were prepared. Paper sheets were milled using a mixer mill (MM400, Retsch GmbH, Haan, Germany) and then mixed with a definite amount of sucrose and norfloxacin. The physical mixture obtained (paper/norfloxacin/sucrose) contains 10 *w*/*w*% norfloxacin and 20 *w*/*w*% sucrose.

For quantitative analysis, physical mixtures without sucrose were prepared. Again, paper sheets were milled and then mixed with a specific amount of norfloxacin. The blends of paper and norfloxacin obtained contain 2.5, 5, 10, 15, 20, and 25 *w*/*w*% norfloxacin.

#### 2.2.3. smartFilms Loaded with Norfloxacin

Norfloxacin-loaded smartFilms and smartFilm granules were produced as described previously [11,15] with minor modifications. First, paper sheets with a size of 5 × 5 cm^2^ and an individual mass of approximately 200 mg were prepared. Second, a solution containing 2.5 mg/mL norfloxacin was prepared by dissolving norfloxacin in a 1:1-mixture of acetone and ethanol. Then, 0.5 mL of the norfloxacin solution was loaded onto the paper sheets by using an automatic micropipette. The paper sheets were left to dry under ambient conditions. To prepare smartFilms loaded with 20 mg (i.e., 10 *w*/*w*%) of norfloxacin this process was repeated 16 times.

#### 2.2.4. Granulation of Norfloxacin-Loaded smartFilms

After drying, the smartFilms loaded with norfloxacin were used to produce granules. For that, the smartFilms were first dry milled for one minute using a knife mill (Moulinex DP8108, Groupe SEB Deutschland GmbH, Frankfurt, Germany). Then, sucrose was added to obtain a mixture containing 20 *w*/*w*% sucrose. In order to dissolve the sucrose and to increase the density of the blend, purified water was sprayed on top of the milled mixture. The blend was grinded for one minute and transferred to a plastic sieve. While shaking the mixture for a period of 3–8 min at 300 rpm (universal shaker SM-30 control, Edmund Bühler GmbH, Bodelshausen, Germany), additional purified water was added to ensure moisturization of the entire mixture. The wet norfloxacin-loaded granules made from smartFilms were then dried in an oven (UN 30, Memmert GmbH + Co. KG, Schwabach, Germany) for 30 min at 120 °C. Finally, the resulting granules were sieved using a mesh size of 2.8 mm (Retsch GmbH, Haan, Germany) to obtain a particle size fraction ≤ 2.8 mm.

#### 2.2.5. Production of Tablets

All samples (except the pure samples, Section 2.2.1) were compressed into flat-faced bevel-edged tablets by using a single punch tablet press (EK0, Korsch GmbH, Berlin, Germany). A compression force of 30 kN was applied and the press was equipped with a 10 mm flat-faced punch (Ritter Pharma-Technik GmbH, Stapelfeld, Germany). The resulting samples had a diameter of 10 mm and a thickness of approximately 0.7–1.3 mm. All tablets were prepared and investigated in triplicate.

In order to determine the influence of wet granulation on the crystallinity of norfloxacin in the formulations, tablets directly made from smartFilms (non-granulated tablets) and tablets made from granules (granulated tablets) were studied. In total, seven sample types were investigated: (i) pure norfloxacin, (ii) pure sucrose, (iii) unloaded paper (i.e., blank smartFilms), (iv) physical mixtures (paper/norfloxacin/sucrose, 10 *w*/*w*% norfloxacin, 20 *w*/*w*% sucrose), (v) physical mixtures for quantitative analysis (paper/norfloxacin, varying mass ratio), (vi) non-granulated smartFilm tablets and (vii) granulated smartFilm tablets.

### 2.3. Solid-State Characterization

#### 2.3.1. X-ray Powder Diffraction

In order to investigate the crystalline state of the different samples an X-ray powder diffractometer (X’Pert Pro MDP, PANalytical/Philipps BV, Netherlands) was used at room temperature. The device was equipped with a Cu-anode (K_α_ radiation, λ = 1.7903 Å) and the voltage and current were set to 40 kV and 35 mA, respectively. Diffraction patterns were collected from 2θ = 10° to 2θ = 55° with a speed of 0.03°/s and a step size of 0.026°. The diffraction patterns of norfloxacin and sucrose were recorded from the bulk materials, whereas the unloaded paper, physical mixtures, and granulated and non-granulated norfloxacin-loaded smartFilms were investigated as tablets.

#### 2.3.2. Terahertz Time-Domain Spectroscopy

For the THz TDS measurements, a fiber-coupled system was used in transmission mode. To minimize the absorption of THz radiation due to the ambient air humidity, all measurements were performed under a nitrogen atmosphere. Details about the THz TDS system and the technique can be found in references [39,40]. The THz beam path was formed by using four off-axis-parabolic mirrors and the samples were placed in the focal plane between the second and the third mirror. The time-resolved waveform of a THz electromagnetic transient passing through a sample was recorded. Reference measurements were taken by moving the samples out of the THz beam. In total, five measurements each with 100 averages per sample were recorded. To calculate the absorption coefficient spectra, the THz waveforms were analyzed using the TeraLyzer software [41]. The software is based on algorithms presented in references [42,43] and enables the evaluation of the refractive index, absorption coefficient, and sample thickness simultaneously. The resulting spectral resolution was 0.01 THz.

## 3. Results

**Figure 1:**
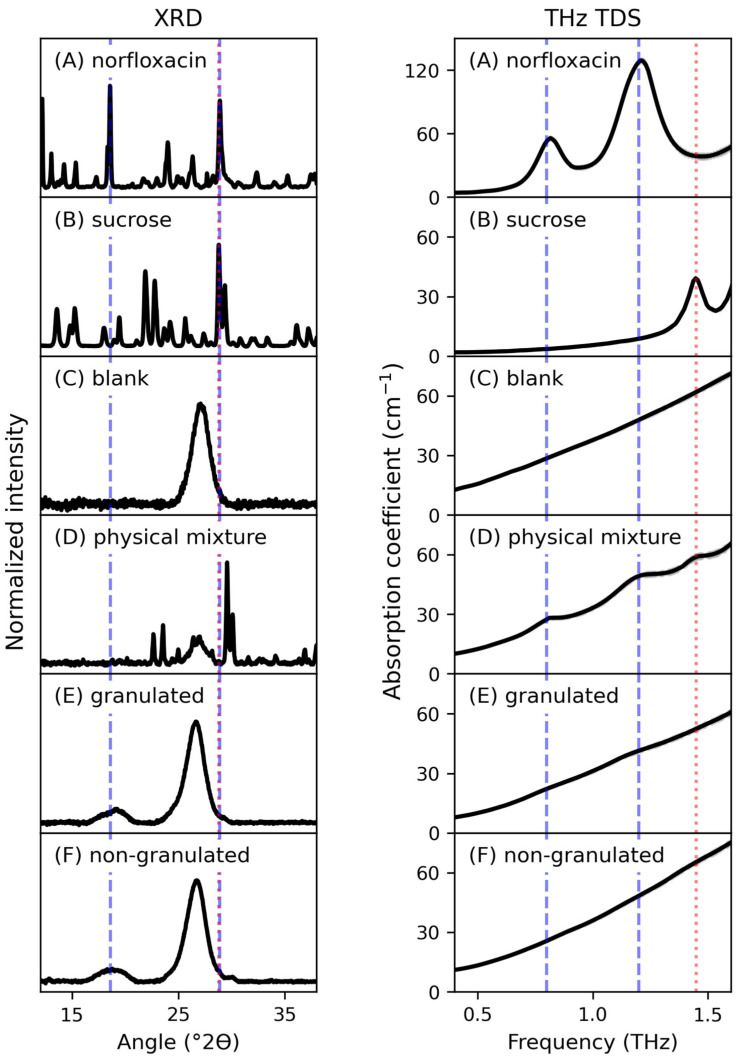
Normalized X-ray powder diffractogram (**left** panel) and THz absorption spectra (**right** panel) of (**A**) crystalline norfloxacin, (**B**) crystalline sucrose, (**C**) blank smartFilm tablet, (**D**) physical mixture tablet containing 20 *w*/*w*% sucrose and 10 *w*/*w*% norfloxacin, (**E**) granulated norfloxacin-loaded smartFilm tablet and (**F**) non-granulated norfloxacin-loaded smartFilm tablet. The horizontal lines mark the most prominent diffraction peaks (panels on the left, XRD) or the characteristic absorption features (panels on the right, THz TDS) of crystalline norfloxacin (dashed blue lines) and sucrose (dotted red line). For clarity, only one diffractogram/spectrum is shown per sample type.

The X-ray powder diffractograms of crystalline norfloxacin, crystalline sucrose, blank smartFilm tablets, physical mixture tablets containing sucrose (20 *w*/*w*%) and norfloxacin (10 *w*/*w*%) as well as the results of the granulated and non-granulated smartFilm tablets are shown in the left panel of Figure 1A–F, respectively. Regarding crystalline norfloxacin (Figure 1A) the two most prominent diffraction features appear at 18.6° and 28.9° (dashed blue lines), whereas for crystalline sucrose (Figure 1B) the most intense peaks can be found at 28.8° (dotted red line). The blank smartFilm tablet (Figure 1C) shows only one broad feature around 27° which can be related to the crystalline cellulose in the paper fibers [44]. This feature can also be found in the diffractograms of the physical mixtures (Figure 1D) together with additional peaks corresponding to crystalline norfloxacin and/or sucrose. However, these features are shifted with regard to the bulk materials and do not represent a linear combination of the peaks of the raw materials which could result from specimen-displacement [45]. Hence, a clear and fast discrimination between crystalline sucrose and norfloxacin in these mixtures can be troublesome. The diffractograms of the granulated and non-granulated smartFilm tablets (Figure 1E,F) show similar patterns compared to the blank tablet, indicating that norfloxacin occurs in an amorphous state in both formulations. Since no reflections corresponding to crystalline sucrose can be detected, one can assume that sucrose also exists in an amorphous state after the granulation. The results obtained by XRD suggest that wet granulation of the loaded paper fibers has no influence on the crystallinity of norfloxacin.

The THz absorption coefficient spectra of crystalline norfloxacin, crystalline sucrose, blank smartFilm tablets, physical mixture tablets containing sucrose (20 *w*/*w*%) and norfloxacin (10 *w*/*w*%) as well as the results of the granulated and non-granulated smartFilm tablets are shown in the right panel of Figure 1A–F, respectively. The corresponding refractive indices are depicted in Figure S1 in the Supporting Material. For crystalline norfloxacin (Figure 1A) two distinctive absorption peaks can be observed in the shown frequency range, one weaker peak at 0.8 THz and one stronger centered around 1.2 THz. In the case of sucrose, one distinct absorption peak at 1.45 THz can be detected in the spectra (Figure 1B). The blank smartFilm tablets (Figure 1C) only show a monotonously rising THz spectra without prominent absorption features, which can be related to the known absorption characteristics of cellulose in the range studied [46]. The THz absorption spectrum of the physical mixture (Figure 1D) reveals the typical absorption peaks of crystalline norfloxacin and sucrose. In comparison to the XRD results, a fast and simple distinction between the spectral features related to crystalline norfloxacin and sucrose is possible in the THz region as the peaks do not overlap. The non-granulated smartFilm tablets (Figure 1F) again only show monotonously rising THz spectra, indicating that norfloxacin exists in an amorphous state in the formulation. In comparison to that, a slightly stronger absorption at the characteristic frequencies of crystalline norfloxacin (dashed blue lines) can be detected for the granulated samples (Figure 1E). This indicates that a small amount of crystalline norfloxacin is present after the wet granulation. Again, no absorption peaks related to crystalline sucrose (dotted red line) can be detected for the granulated samples (Figure 1E), suggesting that sucrose occurs in an amorphous state after the granulation.

**Figure 2:**
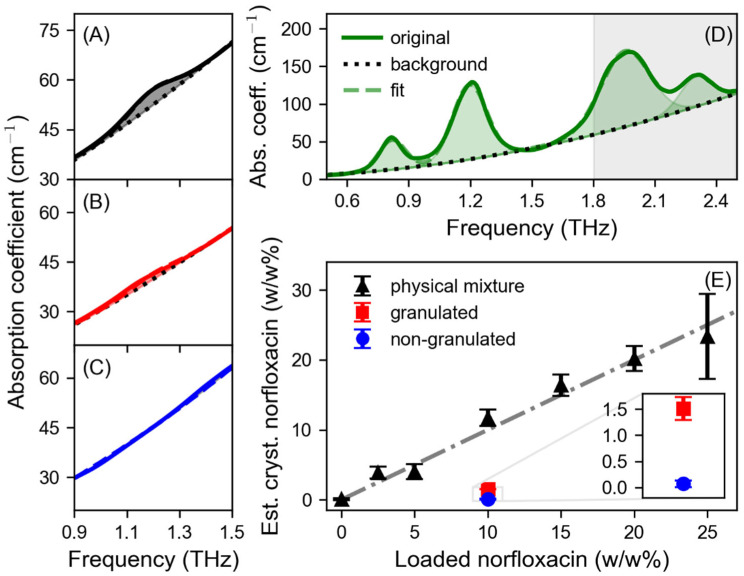
Fitted absorption coefficient (Abs. coeff.) of (**A**) physical mixture tablet containing 10 *w*/*w*% crystalline norfloxacin, (**B**) granulated norfloxacin-loaded smartFilm tablet, (**C**) non-granulated norfloxacin-loaded smartFilm tablet and (**D**) crystalline norfloxacin. The solid lines represent the original spectra, the black dotted lines the fitted background absorption and the dashed lines the fitted spectra. In (**A**–**C**) the spectral region around the most prominent absorption peak in the investigated range is shown and the shaded areas relate to the amount of crystalline norfloxacin in the samples. In (**D**) the shaded areas illustrate the fitted peaks. The grey box indicates the frequency region that was not considered for the analysis of the physical mixtures and smartFilm tablets. (**E**) Estimated crystalline (Est. cryst.) norfloxacin content in physical mixtures, granulated and non-granulated smartFilm tablets. The linear fit regarding the physical mixtures is shown as the grey dashed-dotted line. Inset: estimated crystalline norfloxacin content in granulated (red square) and non-granulated (blue circle) smartFilm tablets. The error bars represent the standard deviation of the assessed crystalline norfloxacin content of the three samples per sample type. Note that a closer look at the determined crystalline content of the granulated and non-granulated tablets shown in the inset in (**E**) reveals that the values clearly differ.

The suitability of THz TDS to quantify the amount of crystalline APIs in mixed formulations has been reported in several studies [11,34,47,48]. Here, a previously described approach was used to determine the percentage of crystalline norfloxacin in the tablets [11]. For this, the spectral features of norfloxacin up to 2.5 THz were considered (Figure 2D). First, the absorption spectrum of crystalline norfloxacin was used to extract the amplitude, frequency position, and width of the characteristic peaks of the API. Therefore, the spectrum was fitted by a sum of a second-degree polynomial to describe the featureless background absorption (Figure 2D, black dotted line), mainly due to scattering, and four Gaussian functions representing the absorption peaks (Figure 2D, green shaded area). The extracted peak parameters are listed in Table 1.

Then, the extracted peak parameters were kept as fixed properties and used to extract the amount of crystalline norfloxacin in the smartFilm tablets. For that, physical mixtures containing a known amount of crystalline norfloxacin (2.5, 5, 10, 15, 20 and 25 *w*/*w*%) were investigated with THz TDS to determine a scaling factor. The absorption coefficient was calculated analogously to the previous samples. In general, the usable spectral bandwidth is limited by the frequency-dependent dynamic range and signal-to-noise ratio of the THz signal [49,50]. All physical mixtures and smartFilm tablets had a larger thickness than the pure norfloxacin sample, leading to a greater absorbance and therefore limiting the frequency range, for which the absorption coefficient could be extracted. In order to ensure a consistent analysis of all samples, only the spectral range between 0.4 and 1.8 THz was analyzed. The frequency region that was not considered for the analysis of the physical mixtures and smartFilm tablets is marked as the grey shaded box in Figure 2D. Furthermore, only the first three absorption peaks of norfloxacin (Table 1) were considered in the analysis. Hence, the following fit function was applied to the absorption spectra:

(1) $$f\left(v\right)=A\overset{3}{\underset{i=1}{\sum }}G_{i}e^{\left(-\frac{{\left(\nu -\nu_{0}^{i}\right)}^{2}}{2\Delta \nu_{i}{}^{2}}\right)}+O\left(2\right)$$

with $G_{i}$, $v_{0}^{i}$ and $\Delta \nu_{i}$ as the amplitude, frequency position and width of the i-th identified peak, respectively. The $O\left(2\right)$-term represents the second-degree polynomial to describe the monotonously rising background absorption, whereas A is the obtained fit coefficient and related to the crystalline norfloxacin content in the mixtures. As an example, the fitted spectrum of a physical mixture containing 10 *w*/*w*% crystalline norfloxacin is shown in Figure 2A. The solid line represents the original spectrum, the black dotted line the fitted background absorption, the dashed line the fitted spectrum and the shaded area relates to the amount of crystalline norfloxacin in the sample. Since the actual content of norfloxacin in the physical mixtures is known, one can use the extracted values of the fit coefficient A to calculate a linear conversion factor between the known and estimated crystalline content. This conversion factor is determined as the slope of the linearly increasing crystalline content in the physical mixtures and enables the assessment of the crystalline norfloxacin content in the samples. Details about the approach used to quantify the amount of crystalline norfloxacin in the samples can be found in reference [11]. The estimation of the crystalline norfloxacin content for the physical mixtures is shown in Figure 2E (black triangles). The grey dashed-dotted line corresponds to the linear fit.

Following the definition of the International Council for Harmonisation of Technical Requirements for Pharmaceuticals for Human Use (ICH), the detection limit and quantitation limit of the used procedure can be expressed as,

(2) $$DL=\frac{3.3\sigma }{S},$$

(3) $$QL=\frac{10\sigma }{S},$$

with DL, QL, σ and S as the detection limit, quantitation limit, standard deviation of the response and slope of the calibration curve, respectively [51]. Using the standard deviation of blanks as σ, the resulting DL and QL are 0.46 *w*/*w*% and 1.39 *w*/*w*%, respectively.

In Figure 2B,C, the absorption spectra fitted for a granulated and a non-granulated norfloxacin-loaded smartFilm tablet are depicted, respectively. Again, the solid lines represent the original spectra, the black dotted lines the fitted background absorption, the dashed lines the fitted spectra and the shaded areas relate to the amount of crystalline norfloxacin in the sample. The estimated amount of crystalline norfloxacin in the samples is shown in Figure 2E. Since the non-granulated smartFilm tablets do not show absorption peaks related to crystalline norfloxacin, the estimated amount is less than 0.01 *w*/*w*% (Figure 2E, blue circles) and, thus, below the detection limit. In contrast, the estimated crystalline norfloxacin content is higher in the case of the granulated smartFilm tablets (Figure 2E, red squares), i.e., 1.5 *w*/*w*%. This indicates that norfloxacin partially crystallizes due to the wet granulation.

**Figure 3:**
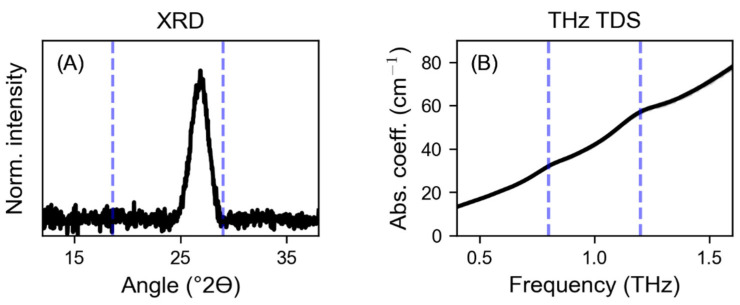
(**A**) Normalized (Norm.) X-ray powder diffractogram and (**B**) THz absorption coefficient (Abs. coeff.) of a physical mixture containing 2.5 *w*/*w*% norfloxacin. The dashed blue horizontal lines mark the position of the most prominent diffraction features (**A**, XRD) or the characteristic absorption peaks (**B**, THz TDS) of crystalline norfloxacin (see panels (**A**) in Figure 1).

To check whether the THz spectrometer used was more sensitive to crystalline norfloxacin, the physical mixture containing only 2.5 *w*/*w*% norfloxacin was additionally investigated by XRD. The corresponding XRD pattern and THz absorption spectrum are shown in Figure 3A,B, respectively. No reflections related to crystalline norfloxacin can be found in the X-ray diffractogram (Figure 3A), indicating that a loading of 2.5 *w*/*w*% is already below the detection limit of the technique. In the THz spectrum, two weak absorption peaks corresponding to crystalline norfloxacin can be identified (Figure 3B). These results support the assumption that, when comparing the two devices used, THz TDS seems to be more sensitive than XRD in detecting low amounts of crystalline norfloxacin in such formulations.

## 4. Discussion

The industrial tablet manufacturing process requires an adequate flowability of the excipients [52]. To overcome the poor flowability of solids (e.g., smartFilm cutouts) their transfer into granules by wet granulation has been extensively studied [15]. However, during this process, the ingredients are exposed to the granulation liquid (i.e., water), which can lead to crystallization [18,19] and subsequently affect the solubility of an API and its bioavailability [7]. Therefore, to determine whether the excipients undergo a phase transformation, quality control and crystallinity assessment are required during the manufacturing process [20]. Regarding the granulated smartFilm tablets, an increased amount of crystalline norfloxacin exceeding the detection limit can be found compared to the non-granulated samples. This indicates that wet granulation of the norfloxacin-loaded smartFilms can lead to partial crystallization of the API in the formulations, which is in accordance with previously reported data regarding the stability of other amorphous drugs under humid conditions [19,53,54]. However, the underlying crystallization mechanisms are still not fully understood because of the complexities of nucleation and crystal growth [55]. It is assumed that the humidity primarily affects the molecular mobility and in particular the α-relaxation, which is, in turn, connected with the translational and rotational diffusion of molecules. Temperature can affect the thermodynamic driving forces in crystallization processes as well as the molecular mobility (reflected in fragility) [19].

In this work, THz TDS was sensitive enough to detect minor changes in the crystallinity, even though the majority of norfloxacin was amorphous. In contrast, such slight changes were not detectable using XRD. This illustrates, when comparing the two devices used, that THz TDS has a lower detection limit than XRD in terms of crystalline norfloxacin. In general, THz TDS cannot provide the same amount of information as XRD for a thorough crystal structure determination, i.e., the unit cell dimensions, but can be used as a fast tool for screening materials and identifying polymorphic forms [21]. Norfloxacin exhibits strongly pronounced absorption features, which is not the case for all relevant APIs. For drugs with weaker absorption features in the THz range, the detection of the crystalline form within the formulation can be more limited, so that XRD still fulfills its role as a standard method for solid-state characterization [20]. However, in certain cases, THz TDS is more sensitive and can provide deeper insights into pharmaceutical formulations. Different studies underlined its potential to detect small amounts of crystalline APIs, which were below the detection limit of other techniques, such as XRD [36,56]. Moreover, it offers general advantages, for instance, no need for ionizing radiation, none or minimal sample preparation, no preferred orientation effects and a fast analysis [21]. 

Furthermore, THz TDS seems to be more suitable to investigate the solid-state of norfloxacin in the formulations studied in this work. A clear distinction between crystalline norfloxacin and sucrose is possible in the THz spectra, whereas the most prominent peaks in the corresponding XRD patterns partly overlap. Additionally, in the diffractograms of physical mixtures, these reflection patterns do not occur as the sum of the individual compounds. Instead, the reflections appear with deviating angles, which requires a more complex and troublesome analysis of the XRD data. For THz TDS, the distinction between the two crystalline compounds is possible, even though the spectra were recorded at room temperature. By performing THz TDS at cryogenic temperatures, narrower and more pronounced absorption peaks are expected, which could allow for even more precise analysis [29]. 

## 5. Conclusions

Various factors can influence the stability and dissolution rate of an amorphous API within the manufacturing process, e.g., the addition of the granulation liquid during the wet granulation procedure. Therefore, quality control including a solid-state characterization of pharmaceutical formulations is essential. In this work, the solid-state analysis was performed using XRD and THz TDS. A comparison of the results obtained shows that XRD was unable to detect differences in the crystalline state before and after the granulation, whereas THz TDS revealed a partial crystallization of norfloxacin in the granulated samples. Additionally, the distinction between the crystalline ingredients was made simpler by THz TDS. Our results show that THz TDS can not only be considered a complementary technique to other established methods, such as XRD, but also an advantageous tool to study the crystallinity of certain APIs in pharmaceutical formulations.

Recent data provide evidence that granulated smartFilm tablets can be utilized as a simple, efficient formulation strategy for the improved solubility and enhanced bioactivity of norfloxacin. Nevertheless, these results suggest that future work should focus on providing a detailed picture of the interactions between the paper matrix and the API. Moreover, the influence of the paper matrix and granulation parameters on the physical stability as well as the pharmaceutical and biopharmaceutical properties of the API should be studied. These findings could lead to an improved granulation process and the production of effective smartFilm tablets that allow an improved bio efficacy of poorly water-soluble APIs.

## 6. Patents

C.M.K. has patent #Cellulose fibre-based support matrices for layered products for oral and peroral application and their preparation (EP17000074.9A) pending to Hoffmann & Sommer und Co KG GmbH.

## Figures and Tables

**Table 1:** Extracted Gaussian peak properties for the individual absorption peaks of crystalline norfloxacin including their amplitude (*G*), frequency position (*v*_0_) and width (Δ*v*).

Peak	*G* (cm^−1^)	*v*_0_ (THz)	Δ*v* (THz)
1	39.32	0.82	0.06
2	99.11	1.20	0.08
3	100.56	1.96	0.12
4	40.00	2.30	0.08

## Data Availability

Data will be made available upon reasonable request.

## Supplementary Materials

The following supporting information can be downloaded at: https://www.mdpi.com/article/10.3390/pharmaceutics15071786/s1, Figure S1. THz refractive index of (A) crystalline norfloxacin, (B) crystalline sucrose, (C) blank smartFilm tablet, (D) physical (phys.) mixture tablet containing 20 *w*/*w*% sucrose and 10 *w*/*w*% norfloxacin, (E) granulated norfloxacin-loaded smartFilm tablet and (F) non-granulated norfloxacin-loaded smartFilm tablet. For clarity, only one spectrum is shown per sample type.
