# Supplementary material for: Terahertz Spectroscopy for Non-Destructive Solid-State Investigation of Norfloxacin in Paper Tablets after Wet Granulation
Source: Pharmaceutics. 2023 Jun 21;15(7):1786. doi: 10.3390/pharmaceutics15071786 (PMC10386691; doi:10.3390/pharmaceutics15071786)
Supplement: Supplementary file 1 [file pharmaceutics-15-01786-s001.zip › pharmaceutics-2441106-supplementary.pdf]

Supplementary Material

Terahertz spectroscopy for non-destructive solid-state investigation of norfloxacin in paper tablets after wet granulation

Lara Heidrich[1], Ayat Abdelkader[2,3], Jan Ornik[1], Enrique Castro-Camus[1], Cornelia M. Keck[2], and Martin Koch[1]

[1]Department of Physics and Material Sciences Center, Philipps-Universität Marburg, Renthof 5, 35032 Marburg, Germany
[2]Department of Pharmaceutics and Biopharmaceutics, Philipps-Universität Marburg, Robert-Koch-Str. 4, 35037 Marburg, Germany
[3]Assiut International Center of Nanomedicine, Al-Rajhi Liver Hospital, Assiut University, Assiut 71515, Egypt

Figure S1

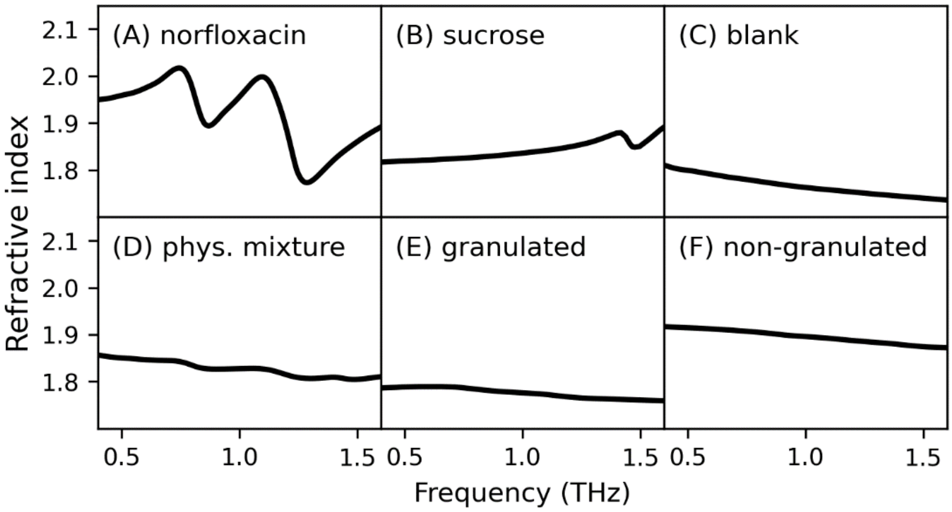

Figure S1. THz refractive index of (A) crystalline norfloxacin, (B) crystalline sucrose, (C) blank smartFilm tablet, (D) physical (phys.) mixture tablet containing 20 w/w% sucrose and 10 w/w% norfloxacin, (E) granulated norfloxacin-loaded smartFilm tablet and (F) non-granulated norfloxacin-loaded smartFilm tablet. For clarity, only one spectrum is shown per sample type.
